# Risk Assessment of Two Insecticides on *Encarsia formosa*, Parasitoid of Whitefly *Bemisia tabaci*

**DOI:** 10.3390/insects9030116

**Published:** 2018-09-11

**Authors:** Zhan He, Yuan Liu, Lei Wang, Qiu Guo, Shaukat Ali, Xiao-Sheng Chen, Bao-Li Qiu

**Affiliations:** 1 Key Laboratory of Bio-Pesticide Innovation and Application, South China Agricultural University, No. 483, Wushan Rd., Tianhe, Guangzhou 510640, China; happy-hezhan@163.com (Z.H.); LYuan@stu.scau.edu.cn (Y.L.); aliscau@scau.edu.cn (S.A.); 2 Engineering Technology Research Center of Agricultural Pest Biocontrol, South China Agricultural University, No. 483, Wushan Rd., Tianhe, Guangzhou 510640, China; wangleiscau@163.com (L.W.); guo_qiu@163.com (Q.G.); 3 College of Forestry and Landscape Architecture, South China Agricultural University, No. 483, Wushan Rd., Tianhe, Guangzhou 510640, China

**Keywords:** aphelinid parasitoid, whitefly, abamectin, imidocloprid, side effect, IPM

## Abstract

The assessment of acute toxicity to insect natural enemies is very important for insecticide selection used within integrated pest management (IPM). The acute toxicity of abamectin and imidacloprid against *Encarsia formosa*, a parasitoid of *Bemisia tabaci*, was investigated. Abamectin had a high toxicity risk to *E. formosa*, while imidacloprid showed a medium toxicity risk. When treated with the lethal concentration 30 (LC_30_) of abamectin, the dwelling time of *E. formosa* in *B. tabaci* infested-plant-area (IPA) was significantly lower than in non-infested plant areas (non-IPA). In addition, the frequency of *E. formosa* entering into the two areas was not significantly different in the LC_10_ and LC_30_ treatments. Within the IPA, LC_10_, and LC_30_ treatments decreased the dwelling time and entering frequency of parasitoid significantly. For imidacloprid treatments, *E. formosa* stayed a longer time in the non-IPA than in the IPA when treated with LC_30._ The frequency of *E. formosa* entering into the two areas was only slightly different in the LC_1_, LC_10_, and LC_30_ treatments. Within the IPA, LC_10_ and LC_30_ treatments were significantly decreased in the dwelling time and the entering frequency of *E. formosa.* The results indicate that abamectin and imidacloprid have high or medium acute toxicity against *E. formosa* and a negative sublethal effect on its searching behaviour.

## 1. Introduction

The whitefly *Bemisia tabaci* (Gennadius) (Hemiptera: Aleyrodidae) is a global agricultural pest [1,2]. This whitefly pest causes serious damage to hundreds of crops directly by sucking phloem sap, and indirectly by causing sooty mould growth via honeydew secretion and, most importantly, by the transmission of numerous plant viruses [3,4,5,6].

Pesticide application still remains the main approach for *B. tabaci* management but the overuse of chemical pesticides has resulted in strong chemical resistance outbreaks for this whitefly pest [7,8]. Therefore, integrated pest management (IPM) is considered a more sustainable approach for the control of *B. tabaci* and, as a key part of IPM, biological control with parasitoids plays an important role in sustainable insect pest control [9]. To date, more than 50 species of parasitoids of *B. tabaci* have been described, among which *Encarsia formosa* Gahan (Hymenoptera: Aphelinidae) is an important, dominant parasitoid species, and it has been reported as an efficient biological agent in whitefly control in many regions of the world [9,10,11,12,13,14].

Insecticide application within an IPM programme should be toxic to target pests and non-toxic to their natural enemies [15,16,17,18]. However, many insecticides that are effective against target pests are also harmful to natural enemies, reducing the effectiveness of biological control [14,19,20]. Therefore, it is essential to evaluate the impact of insecticides on natural enemies. The evaluation of the acute toxicity (i.e., lethal effect) can provide partial assessment concerning the potential effects of pesticides to the exposed non-target organisms [21,22], while sublethal effects of insecticides on natural enemies may ultimately cause a decrease in their efficiency as biological control agents. Therefore, to completely assess the effects of an insecticide on a natural enemy, the risk-assessment should include acute toxicity as well as sub-lethal and chronic effects [23,24]. Based on these findings, the selection of appropriate insecticides that have a less negative impact on a given specific natural enemy is critical in the development and success of IPM of invertebrate pests [25].

Insecticides including abamectin and imidacloprid have been widely used to control *B. tabaci* in many countries [10,26,27,28,29]. The acute toxicities and sublethal impacts of these two insecticides have been evaluated on many parasitoid natural enemies [10,22,30,31,32,33], but the negative impacts of abamectin and imidacloprid on the biology and host searching behaviour of different developmental stages of *E. formosa*, have been little investigated [10,14,34].

In this study, the acute toxicity of abamectin and imidacloprid to different developmental life stages of *E. formosa* as well as their sublethal effects on the parasitoid’s host searching behaviour were assessed. The results are expected to ameliorate the sustainable control of *B. tabaci* in IPM programmes.

## 2. Materials and Methods

### 2.1. Plants, Insects and Insecticides

Seeds of cotton *Gossypium hirsutum* L. (Malvales: Malvaceae) Luman No. 32 were produced by the Cotton Research Center, Shandong Academy of Agricultural Sciences, Jinan, China. Healthy seedlings were cultivated within plastic pots (Φ 20 cm) in screened cages and grown to approximately 30 cm in height before being used in experiments. The cotton plants were not exposed to any pests or pesticides. Two to three smooth and health leaves per plant were selected for experimental tests.

The tobacco whitefly, *Bemisia tabaci* MEAM1 (Middle East Asia Minor 1 cryptic species, formerly B biotype), was originally collected from the training farm of South China Agricultural University (SCAU), Guangzhou in 2005, and maintained on cotton plants in a separated glasshouse in SCAU under ambient conditions. The parasitoid *E. formosa* was first collected from Beijing Academy of Agriculture and Forestry Sciences in 2008, and the population was maintained on cotton plants with *B. tabaci* as the host in separated cages.

The insecticides abamectin (95% certified purity) and imidacloprid (97% certified purity), were obtained from Guangxi Antai Chemicals Co. Ltd., (Nanning, China). The stock solutions of both insecticides (1000 mg/L and 5000 mg/L respectively) were prepared by using acetone as solvent. Five different concentrations of each insecticide (2, 4, 8, 16, 32 mg/L for abamectin and 20, 40, 80, 160, 320 mg/L for imidacloprid) were prepared through serial dilutions for the toxicity tests. Double distilled water (ddH_2_O) was used for the dilutions and as control.

All the bioassays were carried out under controlled environmental conditions at 26 ± 1 °C, 70 ± 10% R.H, photoperiod of 14:10 (L:D), and light intensity of 3000Lx. Artificial climate chambers (RXZ-560, Jiangnan Instrument Factory, Ningbo, China) were used for maintaining the controlled environmental conditions.

### 2.2. Toxicity to Parasitoid Adults

The toxicity of abamectin and imidacloprid to *E. formosa* adults was investigated by exposing adults to dry residues [14,35]. Glass tubes (1.5 cm diameter × 10 cm length) were filled with different insecticide concentrations or ddH_2_O (control). The insecticidal solution was poured out after 10 s and the tubes inverted in order to dry them out. Fifteen *E. formosa* adult wasps (age < 24 h) were then released into each tube for different time intervals (12, 24, 48, 72 and 96 h). The adult wasps were supplied with 15% honey solution drops on the tube wall and the tubes were sealed with Parafilm^®^ (Bemis Company, Inc., Neenah, WI, USA). The mortality of *E. formosa* adults was recorded every 12 h. The concentrations LC_1_, LC_10_, LC_30_ and LC_50_ of both insecticides were calculated through probit analysis (see Data analysis). Each set of treatments was repeated 3 times, with a total of 45 parasitoids tested for each time period.

### 2.3. Toxicity to Parasitoid Pre-Pupae and Pupae

The toxicity of abamectin and imidacloprid to the pre-pupa and pupa of *E. formosa* was investigated by topically exposing the insect via the dipping method [16,36]. Approximately 40 *B. tabaci* female adults were released onto healthy cotton leaves inside leaf cages (5 cm diameter × 3 cm height) for 24 h oviposition, after which all the adults were removed. When the whitefly offspring developed through to 3rd instar nymphs (5–6 days after egg hatching), five *E. formosa* females (2 day old) were released into the leaf cage to parasitize the nymphs for a 12 h period and then removed. When the parasitoids had developed to pre-pupal or pupal stage, their numbers were recorded on each leaf, and unparasitized *B. tabaci* nymphs were removed. The individual leaves with parasitized *B. tabaci* nymphs were then immersed into different insecticide concentrations or ddH_2_O (control) for 10 s, after which the plants were cultured in an artificial climate chamber until all the parasitoids either developed into adults or died. The numbers of adults emerging were recorded daily. The LC_1_, LC_10_, LC_30_ and LC_50_ concentrations of both pesticides were calculated through probit analysis (see Data analysis). Each set of treatments was repeated 3 times, with a total of 120 pre-pupae or pupae tested for each wasp lifestage.

A stereo microscope (SZ45-BL3, Sunny Optical Technology (Group) Co. Ltd., Ningbo, China) was used to observe the insects during the experiments.

### 2.4. Sublethal Effects on Parasitoid Searching Behaviour

A four-armed olfactometer was used for these tests [35]. The olfactometer was divided into 4 areas of equal size. Each area was connected with a flow meter, a humidifying bottle, an air filtering bottle and an odour source bottle. The air flow of each area was controlled at 120 mL/min. The four-arm was surrounded by a shade but had a light on the top of it, so that the insects can only be exposed to light from the top.

Adults of *E. formosa* (age < 24 h) were obtained by collecting pupae on infested cotton leaves into petri dishes with honey solution (15%) provided as food. These newly emerged adults were not exposed to whitefly hosts. Before initiating experiments, 24 h-old *E. formosa* adults were individually treated with sublethal concentrations, calculated from the toxicity experiments to adults, of abamectin (LC_1_ = 0.637 mg/mL; LC_10_ = 1.643 mg/mL; LC_30_ = 3.262 mg/mL) or imidacloprid (LC_1_ = 4.968 mg/mL; LC_10_ = 16.470 mg/mL; LC_30_ = 40.262 mg/mL) for 1h by using the residual film method [14]. The control group of *E. formosa* adults was treated with ddH_2_O. When the experiments began, two healthy cotton plants were placed into 2 odour source bottles named as “non-infested plant area”, while cotton plants with 3rd and 4th instar *B. tabaci* nymphs on their leaves were placed in the other 2 odour source bottles named as “infested plant area”. All the plants used in the experiments were of the same age. One individual *E. formosa* wasp treated as above was released into the four-armed olfactometer through the central hole. The number of times that the *E. formosa* wasp entered into either the non-infested plant area or infested plant area (taken as a dwelling time of more than 5 s), and their final dwelling time in each area was recorded. Each parasitoid was observed for 600 s. The four-armed olfactometer was cleaned with ethanol followed by ddH_2_O after every individual parasitoid test. Each treatment was repeated 3 times with 30 parasitoids tested each repetition.

### 2.5. Data Analysis

The sublethal concentrations (LC_1_, LC_10_, LC_30_ and LC_50_), 95% confidence intervals, relative factors and safety factors of both insecticides were calculated through linear regression. The safety of the two insecticides to *E. formosa* was evaluated according to “Test guidelines on environmental safety assessment for chemical pesticides” [37], and the toxicity risk grades were divided into 5 levels (Table 1) [37].

The differences in *E. formosa* dwelling time, entering frequency in whitefly infested AND non-infested plant areas treated with the same lethal concentration were analyzed using *t*-test. While the differences of *E. formosa* dwelling time, entering frequency in the same host plant areas (whitefly infested OR non-infested plant areas) among different lethal concentrations were compared using Duncan’s test SPSS 17.0 (International Business Machines Corp., Armonk, NY, USA).

## 3. Results

### 3.1. Toxicity to Parasitoid Adults

The LC_30_ and LC_50_ values of abamectin and imidacloprid to the 12, 24, 48, 72 and 96 h old *E. formosa* adults are listed in Table 2 and Table 3 respectively. Results revealed that the LC_30_ and LC_50_ value of both insecticides to *E. formosa* adults increased with the longevity of the parasitoid, indicating the relatively higher chemical tolerance of older adult wasps than the younger adults (Table 2 and Table 3).

The safety factors of abamectin to *E. formosa* adults of different ages ranged from 0.093 to 0.449 (Table 4), falling between the toxicity limit of 0.05 to 0.5 (Table 1), while the safety factors of imidacloprid ranged from 1.311 to 3.097 (Table 4), falling between the toxicity limit of 0.5 and 5 (Table 1). The results indicate that abamectin has a high toxicity risk to adult parasitoids while imidacloprid has a medium toxicity risk to adult parasitoids.

### 3.2. Toxicity to Parasitoid Pre-Pupae and Pupae

Analysis of the results revealed that, the LC_50_ of abamectin to the pre-pupa and pupa of *E. formosa* was 4.395 and 4.474 mg/L, while the LC_30_ was 3.029 and 2.524 mg/L respectively (Table 5). The safety factors calculated for pre-pupa and pupa were 0.220 and 0.224, both of them were between 0.05 to 0.5, which indicates a high toxicity risk to the pre-pupa and pupa of *E. formosa* according to risk grades (Table 6). By contrast, the LC_50_ of imidacloprid on pre-pupa and pupa were 22.211 and 30.781 mg/L, respectively. The LC_30_ values of imidacloprid were 8.877 and 16.990 mg/L to the pre-pupa and pupa (Table 7), and the safety factors were 0.667 and 0.993, respectively, indicating that imidacloprid has a medium toxicity risk to the pre-pupa and pupa of *E. formosa* (Table 6).

### 3.3. Sublethal Effects on Parasitoid Searching Behaviour

**Figure 1:**
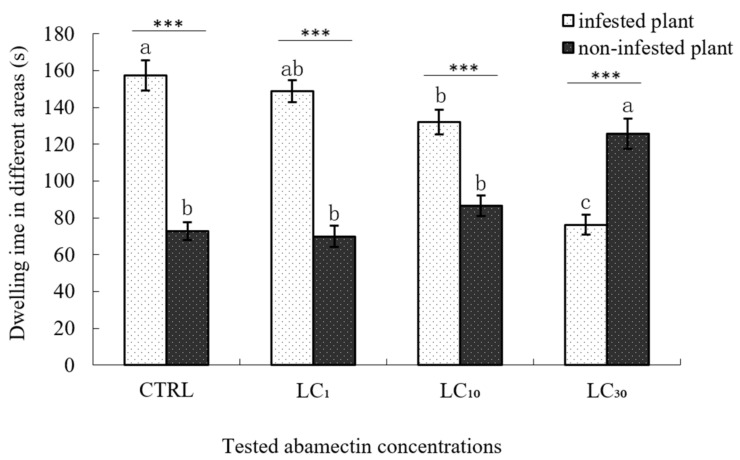
Dwelling time of *Encarsia formosa* in different plant areas treated with abamectin. Note: *** *p* < 0.001 (significantly different between the two groups, *t*-test). The same letter over the bars of infested plant or non-infested plant areas indicates no significant differences between the various concentrations (Duncan’s multiple range test, α < 0.05). The “infested plant area” means cotton plants in the four-armed olfactometer infested with 3rd or 4th instar *B. tabaci* nymphs, while the “non-infested plants” means healthy cotton plants without whitefly infestation in the four-armed olfactometer.

After treatment with LC_1_ and LC_10_ of abamectin for 1 h using the residual film method, *E. formosa* wasps did not change their choice preference, because their dwelling times in the *B. tabaci* infested plant areas were both significantly longer than their dwelling times in the non-infested plant areas (148.62 s vs. 69.90 s in LC_1_ experiment; 131.97 s vs. 86.51 s in LC_10_ experiment). However, when the concentration of abamectin increased to LC_30_, the dwelling time spent by *E. formosa* adults in the area with non-infested plants was significantly longer than the dwelling time in the area with *B. tabaci* infested plants (125.73 s vs. 76.21 s), which indicates that the higher concentration of abamectin (LC_30_) reduced the searching behaviour of *E. formosa*. When compared with the control, the dwelling time of *E. formosa* adults in *B. tabaci* infested plant areas of LC_1_ treatment had no significant difference, while the dwelling time of LC_10_ treatment was significantly lower and LC_30_ treatment was the lowest. While for the dwelling time in the non-infested plant area, no significant differences were found between the control, LC_1_ and LC_10_ treatments, but the dwelling time of LC_30_ treatment was significantly longer (Figure 1).

**Figure 2:**
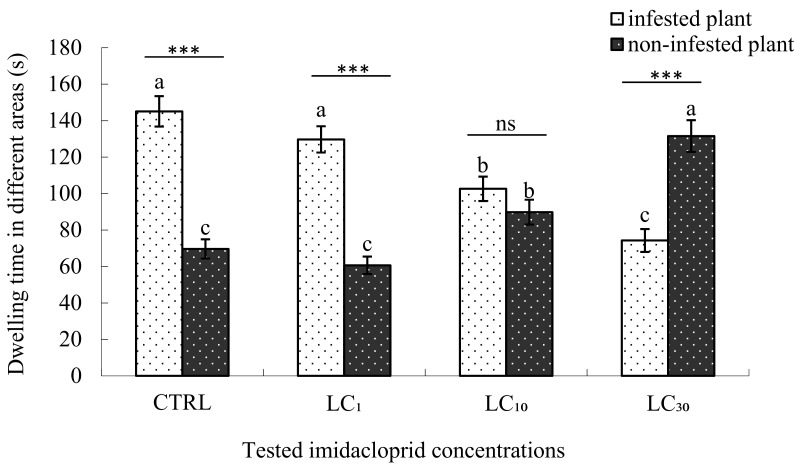
Dwelling time of *Encarsia formosa* in different areas treated with imidacloprid. Note: *** *p* < 0.001 (significantly different between the two groups, *t*-test), ns (not significantly different between the two groups, *t*-test). The same letter over the bars of infested plant or non-infested plant areas indicates no significant differences between the various concentrations (Duncan’s multiple range test, α < 0.05). The “infested plant area” means cotton plants in the four-armed olfactometer infested with 3rd or 4th instar *B. tabaci* nymphs, while the “non-infested plants” means healthy cotton plants without whitefly infestation in the four-armed olfactometer.

Similar to the abamectin tests, the treatment of imidacloprid LC_1_ and LC_10_ did not change the choice preference of *E. formosa* wasps, that is, preferring the *B. tabaci* infested plants over the non-infested plants (129.72 s vs. 60.64 s in LC_1_ experiment; 102.60 s vs. 89.82 s in LC_10_ experiment). However, when the concentration of imidacloprid increased to LC_30_, the dwelling time of *E. formosa* in the non-infested plant area was significantly longer than the dwelling time spent in *B. tabaci* infested plant area (131.55 s vs. 74.26 s). This indicated that when *E. formosa* adults were exposed to the LC_30_ of imidacloprid, an opposite preference of host searching behaviour compared to normal occurred; *E. formosa* could not search for its host as normal when treated with LC_30_ imidacloprid. When compared with the control, the dwelling time of *E. formosa* adults in the *B. tabaci* infested plant area of LC_1_ treatment had no significant difference, while the dwelling time of LC_10_ treatment was significantly lower and the LC_30_ treatment was the lowest. While for the dwelling time in the non-infested plant area, no significant differences were found between the control and LC_1_ treatment, but the dwelling time of LC_10_ treatment was significantly longer and the LC_30_ treatment was the longest (Figure 2).

**Figure 3:**
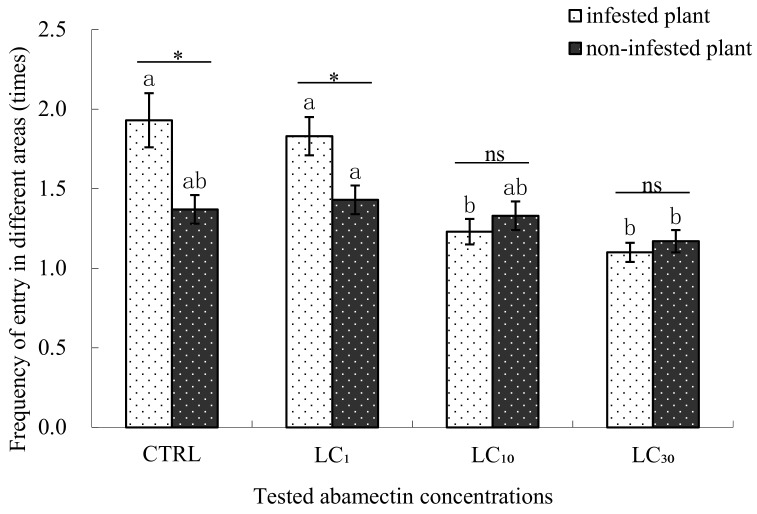
The frequency of *Encarsia formosa* entering different areas when treated with abamectin. Note: * *p* < 0.05 (significantly different between the two groups, *t*-test), ns (not significantly different between the two groups, *t*-test). The same letter over the bars of infested plant or non-infested plant areas indicates no significant differences between the various concentrations (Duncan’s multiple range test, α < 0.05). The “infested plant area” means cotton plants in the four-armed olfactometer infested with 3rd or 4th instar *B. tabaci* nymphs, while the “non-infested plants” means healthy cotton plants without whitefly infestation in the four-armed olfactometer.

In the control and LC_1_ treatment of abamectin, the frequencies that *E. formosa* entered into the *B. tabaci* infested plant areas were relatively higher than the frequencies of entry into the non-infested plant areas (1.93 and 1.83 times vs. 1.37 and 1.43 times respectively), whereas in the LC_10_ and LC_30_ treatments, the frequencies that *E. formosa* entered into the *B. tabaci* infested plant areas were less than that of entering into non-infested plant areas, but there was no significant differences between the two concentration treatments (1.23 and 1.10 times in *B. tabaci* infested plant area vs. 1.33 and 1.17 times in non-infested plant area). When compared with the control, the frequency that *E. formosa* entered into the *B. tabaci* infested plant area of LC_1_ treatment had no significant difference, while the frequencies of LC_10_ and LC_30_ treatments were significantly lower. However, for the frequencies of entering into the non-infested plant area, no significant differences were found between the control and LC_1_, LC_10_, and LC_30_ treatments (Figure 3).

**Figure 4:**
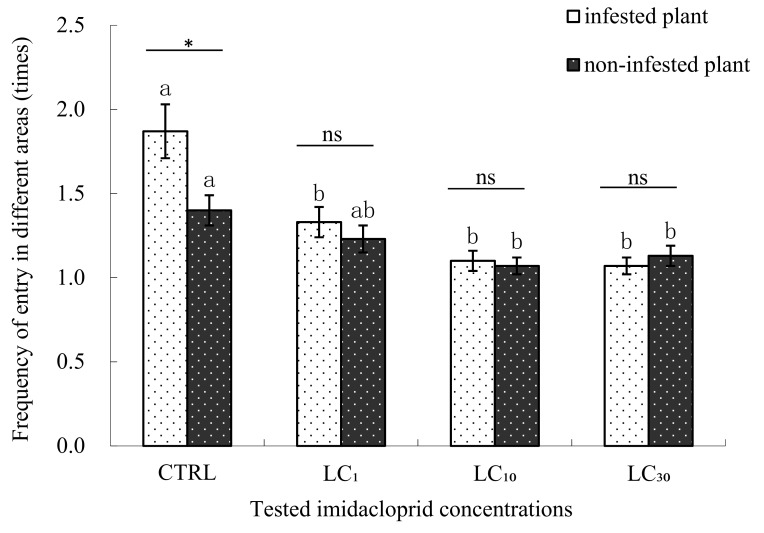
The frequency of *Encarsia formosa* entering different areas when treated with imidacloprid. Note: * *p* < 0.05 (significantly different between the two groups, *t*-test), ns (not significantly different between the two groups, *t*-test). The same letter over the bars of infested plant or non-infested plant areas indicates no significant differences between the various concentrations (Duncan’s multiple range test, α < 0.05). The “infested plant area” means cotton plants in the four-armed olfactometer infested with 3rd or 4th instar *B. tabaci* nymphs, while the “non-infested plants” means healthy cotton plants without whitefly infestation in the four-armed olfactometer.

When treated with LC_1_, and LC_10_ concentrations of imidacloprid, the frequencies that *E. formosa* entered into the *B. tabaci* infested plant areas were only a little more than that of entering into the non-infested plant areas (1.33 and 1.10 times vs. 1.23 and 1.07 times respectively), while in the LC_30_ concentration test, the situation has reversed, the frequency that *E. formosa* entered into the *B. tabaci* infested plant areas were only a little less than that of entering into the non-infested plant areas (1.07 times vs. 1.13 times), but there was no significant differences between the entering frequencies in the LC_1_, LC_10_ and LC_30_ concentration experiments. Similarly, the frequencies that *E. formosa* entered into the *B. tabaci* infested plant area of LC_1_, LC_10_, and LC_30_ treatments were significantly less than the frequency of the control. For the frequencies of entering into the non-infested plant areas, no significant differences were found between the control and LC_1_ treatment but the LC_10_ and LC_30_ treatments were significantly less than the control (Figure 4).

## 4. Discussion

This current research has shown that abamectin has a high toxicity risk against *E. formosa*. This is consistent with the results of Bacci et al. [10], who reported high insecticidal toxicity of abamectin to adults of *Encarsia* sp. (Hymenoptera: Aphelinidae). Abamectin has also been found to be highly toxic against the adults of other parasitoid wasps, such as *Trichogramma chilonis* Ishii (Hymenoptera: Trichogrammatidae), *Bracon nigricans* Szépligeti (Hymenoptera: Braconidae) and *Aphidius gifuensis* (Ashmead) (Hymenoptera: Braconidae) [24,32,33]. However, results of some other studies differ with those of the present study, for example, abamectin was considered harmless to *Agenia spiscitricola* Longvinovskaya (Hymenoptera: Encyrtidae) [38], slightly to moderately toxic to *Trichogramma japonicum* Ahmead (Homenoptera: Trichogrammatidae) [29], and moderately harmful to *Aphytis melinus* DeBach (Hymenoptera: Aphelinidae) [39]. We suggest that the different conclusions regarding the toxicity may be due to different experimental methods (e.g., insecticide residues on leaves and hosts surfaces in some other experiments but on glass surfaces in our experiments; exposure durations in their experiments were 1 h, 24 h, 3 d, but varied in 12, 24, 48, 72, and 96 h in our experiments; the ages of the insects used for experiments in their researches were 24–48 h old, or <48 h old, or <3 d old, but <24 h old in our research.) and the parasitoid species investigated.

Results in the current study indicated a medium toxicity risk of imidacloprid to *E. formosa*, and this is similar to the study of Prabhaker et al. [40] with the same insecticide and parasitoid. However, there are also studies that revealed a more severe toxicity of imidacloprid to several species of parasitoids, including *Encarsia* sp. [10], *Encarsia inaron* (Walker) (Hymenoptera: Aphelinidae) [30], *Eretmocerus mundus* Mercet (Hymenoptera: Aphelinidae) [31], *Eretmocerus eremicus* Rose and Zolnerowich (Hymenoptera: Aphelinidae), *A. melinus*, *Gonatocerus ashmeadi* Girault (Hymenoptera: Mymaridae) [40] and *Trichogramma cacoeciae* Marchal (Hymenoptera: Trichogrammatidae) [41]. These different conclusions may again be also due to the experimental methods, insecticide concentrations and the parasitoid species investigated as we mentioned above.

While previous studies investigated the toxicity of insecticides on adults of *E. formosa* without any concern of their age, the current study investigated this biological factor with abamectin and imidacloprid following *E. formosa* emergence. It was found that these two insecticides had different risk levels against *E. formosa* adults, in particular abamectin has a high toxicity risk while imidacloprid has a medium toxicity risk.

In the current study, we investigated the toxicity of abamectin and imidacloprid to the pre-pupa and pupa of *E. formosa*. Here we obtained similar results to the toxicity to the adults: a high toxicity risk of abamectin and a medium toxicity risk of imidacloprid. Harmful effects of abamectin have also been found on the mummy stage of *A. gifuensis* but the mortalities observed were much fewer than when against the adult stage [33]. Mahdavi et al. [22] classified abamectin as slightly harmful when pre-imaginal stages of *Habrobracon hebetor* Say (Hymenoptera: Braconidae) were treated with this chemical. For imidacloprid, Saber [41] found lower toxicity to the larvae, pre-pupae and pupae of *T. cacoeciae* when compared to the adults. The higher toxicity of insecticides to adults than to pre-pupae or pupae of parasitoids is probably due to immature parasitoids developing within host bodies are protected from many pesticides [23,33,42,43]. The mechanism of contrary findings between this study and previous studies requires further investigation, but the sensitivity of different parasitoid species to a given insecticide maybe one of the biological factors playing a role [22,44]. Different methods employed in experiments for adults (insecticide on glass surface in our experiments) and pre-pupae and pupae (insecticide on leaf surface in our experiments) may also lead to differences in a parasitoid’s response to an insecticide. The side effects of these two insecticides on natural enemies might be waved in field comparing to that in the laboratory. For example, when applied in the field, abamectin was recorded to be not harmful to parasitoids on Java potato [45], but imidacloprid has negative effects on the parasitism and population dynamics of aphid parasitoids [46,47].

Sublethal concentrations of pesticides may not only affect the physiological traits of a natural enemy (e.g., lifespan, growth and reproduction), but can also interfere its behaviour in regards to host searching, learning, and communication [20,23,35]. In this study, sublethal concentrations of both insecticides (abamectin and imidacloprid) caused a distinct negative influence on the efficiency of the parasitoids’ host searching ability. Similar results were found for *Neochrysocharis formosa* (Westwood) (Hymenoptera: Eulophidae) [48] and *Anagrus nilaparvatae* (Pang et Wang) (Hymenoptera: Mymaridae) [49] when the parasitoids were treated with sublethal concentrations of imidacloprid. Bethke and Redak [50] found lower parasitism of *E. formosa* on *Bemisia argentifolii* Bellows and Perring (Hemiptera: Aleyrodidae) when the pest nymphs were treated with imidacloprid. When treated with an LC_30_ of abamectin, the host searching behaviour of *E. formosa* was significantly affected, but it was less influenced when treated with the LC_10_ concentration, while for LC_1_ treatment it was slightly affected. The behaviour was also remarkably forced into a reverse trend when treated with LC_1_, LC_10_ and LC_30_ of imidacloprid. The reduction of host searching behaviour in our results can lead to both decreased oviposition and parasitism rate. Pesticides can lead to a reduction in the parasitoids’ sensitivity and response to volatiles from hosts or pest-infested plants [49]. This may be because of the obstruction or destruction of perception and response to related pheromones [51,52], so the searching efficiency is reduced.

## 5. Conclusions

In summary, our results have indicated that abamectin and imidacloprid both have high or medium acute toxicity against *E. formosa* and negative sublethal effects on its host searching behaviour. Therefore, abamectin and imidacloprid should be more cautiously used within IPM programmes against *B. tabaci* as they have been shown to reduce the effectiveness of *E. formosa*. In addition, the residue of these insecticides within the environment (on plants in the soil), as well as other non-target effects combining biotic and abiotic factors to this parasitoid, should be further studied [53]. Discrete precautionary measures should be taken before applying these insecticides or, indeed, some other effective and sustainable methods. Furthermore, products should be evaluated and applied within IPM programmes against *B. tabaci* in their place.

## Figures and Tables

**Table 1:** Definition of the pesticide risk grades to insects.

Toxicity Risk Grade	Safety Factor (SF)
extreme high risk	SF ≤ 0.05
high risk	0.05 < SF ≤ 0.5
medium risk	0.5 < SF ≤ 5
low risk	SF > 5

Note: safety factor = LC_50_ (mg/L)/ield recommended concentration (mg/L).

**Table 2:** Toxicity of abamectin to *Encarsia formosa* adults.

Parasitoid Age (h)	Regression Equation of Toxicity	Correlation Coefficient (r)	LC_30_ (mg/L) (95% Confidence Interval)	LC_50_ (mg/L) (95% Confidence Interval)
12	Y = −0.396 + 1.472X	0.966	0.818 (0.255–1.456)	1.857 (0.891–2.771)
24	Y = −1.829 + 2.541X	0.928	3.262 (0.443–5.839)	5.246 (1.894–11.081)
48	Y = −1.770 + 2.162X	0.973	3.768 (2.805–4.707)	6.587 (5.330–8.078)
72	Y = −2.092 + 2.499X	0.987	4.239 (3.312–5.150)	6.872 (5.695–8.266)
96	Y = −2.439 + 2.560X	0.991	5.598 (4.457–6.740)	8.972 (7.490–10.804)

**Table 3:** Toxicity of imidacloprid to *Encarsia formosa* adults.

Parasitoid Age (h)	Regression Equation of Toxicity	Correlation Coefficient (r)	LC_30_ (mg/L) (95% Confidence Interval)	LC_50_ (mg/L) (95% Confidence Interval)
12	Y = −2.854 + 1.740X	0.976	21.818 (12.965–30.399)	43.669 (31.555–56.3)
24	Y = −3.789 + 2.034X	0.974	40.262 (29.717–50.668)	72.892 (58.593–90.425)
48	Y = −4.441 + 2.301X	0.981	43.818 (30.749–56.726)	81.296 (66.387–99.827)
72	Y = −3.392 + 1.747X	0.983	46.943 (35.828–57.980)	87.470 (68.605–112.343)
96	Y = −3.399 + 1.688X	0.986	50.445 (35.926–65.201)	103.138 (80.635–135.647)

**Table 4:** The toxicity risk grade of abamectin and imidacloprid to *Encarsia formosa* adults.

Pesticide	Parasitoid Age (h)	Field Recommended Concentration (mg/L)	Safety Factor (SF)	Toxicity Risk Grade
abamectin	12	20	0.093	high risk
	24	20	0.262	high risk
	48	20	0.329	high risk
	72	20	0.344	high risk
	96	20	0.449	high risk
imidacloprid	12	33.3	1.311	medium risk
	24	33.3	2.189	medium risk
	48	33.3	2.441	medium risk

**Table 5:** Toxicity of abamectin to the pre-pupa and pupa of *Encarsia formosa*.

Parasitoid Stage	Regression Equation of Toxicity	Correlation Coefficient (r)	LC_30_ (mg/L) (95% Confidence Interval)	LC_50_ (mg/L) (95% Confidence Interval)
pre-pupa	Y = −2.086 + 3.244X	0.951	3.029 (0.386–5.167)	4.395 (1.447–8.493)
pupa	Y = −1.372 + 2.109X	0.990	2.524 (1.730–3.278)	4.474 (3.473–5.532)

**Table 6:** The toxicity risk grade of abamectin and imidacloprid to pre-pupa and pupa of *Encarsia formosa*.

Pesticide	Parasitoid Stage	Field Recommended Concentration (mg/L)	Safety Factor (SF)	Toxicity Risk Grade
abamectin	pre-pupa	20	0.220	high risk
	pupa	20	0.224	high risk
imidacloprid	pre-pupa	33.3	0.667	medium risk
	pupa	33.3	0.993	medium risk

**Table 7:** Toxicity of imidacloprid to the pre-pupa and pupa of *Encarsia formosa*.

Parasitoid Stage	Regression Equation of Toxicity	Correlation Coefficient (r)	LC_30_ (mg/L) (95% Confidence Interval)	LC_50_ (mg/L) (95% Confidence Interval)
pre-pupa	Y = −1.773 + 1.317X	0.931	8.877 (2.822–15.911)	22.211 (11.107–33.026)
pupa	Y = −3.024 + 2.032X	0.951	16.990 (1.210–32.911)	30.781 (6.680–54.553)

## References

[1] Brown J.K., Frohlich D.R., Rosell R.C. (1995). The sweetpotato or silverleaf whiteflies: Biotypes of *Bemisia tabaci* oraspeciesco complex. Annu. Rev. Entomol..

[2] De Barro P.J., Liu S.S., Boykin L.M., Dinsdale A.B. (2011). *Bemisia tabaci*: A statement of species status. Annu. Rev. Entomol..

[3] Berlinger M.J. (1986). Host plant resistance to *Bemisia tabaci*. Agric. Ecosyst. Environ..

[4] Jones D.R. (2003). Plant viruses transmitted by whiteflies. Eur. J. Plant Pathol..

[5] Qiu B.L., Dang F., Li S.J., Ahmed M.Z., Jin F.L., Ren S.X., Cuthbertson A.G.S. (2011). Comparison of biological parameters between the invasive B biotype and a new defined Cv biotype of *Bemisia tabaci* (Hemiptera: Aleyrodidae) in China. J. Pest Sci..

[6] Su Q., Pan H.P., Liu B.M., Chu D., Xie W., Wu Q.J., Wang S.L., Xu B.Y., Zhang Y.J. (2013). Insect symbiont facilitates vector acquisition, retention, and transmission of plant virus. Sci. Rep..

[7] Cuthbertson A.G.S., Buxton J.H., Blackburn L.F., Mathers J.J., Robinson K.A., Powell M.E., Fleming D.A., Bell H.A. (2012). Eradicating *Bemisia tabaci* Q biotype on poinsettia plants in the UK. Crop Prot..

[8] Basit M., Saeed S., Ahmad M., Sayyed A.H. (2013). Can resistance in *Bemisia tabaci* (Homoptera: Aleyrodidae) be overcome with mixtures of neonicotinoids and insect growth regulators?. Crop Prot..

[9] Gerling D., Alomar Ò., Arnò J. (2001). Biological control of *Bemisia tabaci* using predators and parasitoids. Crop Prot..

[10] Bacci L., Crespo A.L.B., Galvan T.L., Pereira E.J.G., Picanco M.C., Silva G.A., Chediak M. (2007). Toxicity of insecticides to the sweetpotato whitefly (Hemiptera: Aleyrodidae) and its natural enemies. Pest Manag. Sci..

[11] Li S.J., Xue X., Ahmed M.Z., Ren S.X., Du Y.Z., Wu J.H., Cuthbertson A.G.S., Qiu B.L. (2011). Host plants and natural enemies of *Bemisia tabaci* (Homoptera: Aleyrodidae) in China. Insect Sci..

[12] Van Lenteren J.C., Van Roermund H.J.W., Sutterlin S. (1996). Biological control of greenhouse whitefly (*Trialeurodes vaporariorum*) with the parasitoid *Encarsia formosa*: How does it work?. Biol. Control.

[13] Van Lenteren J.C. (2000). A greenhouse without pesticides: Fact or fantasy. Crop Prot..

[14] Sugiyama K., Katayama H., Saito T. (2011). Effect of insecticides on the mortalities of three whitefly parasitoid species, *Eretmocerus mundus*, *Eretmocerus eremicus* and *Encarsia formosa* (Hymenoptera: Aphelinidae). Appl. Entomol. Zool..

[15] Cuthbertson A.G.S., Murchie A.K. (2005). European red spider mite—An environmental consequence of persistent chemical pesticide application. Int. J. Environ. Sci. Technol..

[16] Prabhaker N., Morse J.G., Castle S.J., Naranjo S.E., Henneberry T.J., Toscano N.C. (2007). Toxicity of seven foliar insecticides to four insect parasitoids attacking citrus and cotton pests. J. Econ. Entomol..

[17] Bueno A.F., Batistela M.J., Bueno R.C.O.F., Franca-Neto J.B., Nishikawa M.A.N., Filho A.L. (2011). Effects of integrated pest management, biological control and prophylactic use of insecticides on the management and sustainability of soybean. Crop Prot..

[18] Biondi A., Desneux N., Siscaro G., Zappalà L. (2012). Using organic-certified rather than synthetic pesticides may not be safer for biological control agents: Selectivity and side effects of 14 pesticides on the predator *Orius laevigatus*. Chemosphere.

[19] Cuthbertson A.G.S., Murchie A.K. (2006). The environmental impact of an orchard winter wash and early season pesticide applications on both a beneficial and a pest mite species in Bramley apple orchards. Int. J. Environ. Sci. Technol..

[20] Cloyd R.A., Bethke J.A. (2011). Impact of neonicotinoid insecticides on natural enemies in greenhouse and interiorscape environments. Pest Manag. Sci..

[21] Walthall W.K., Stark J.D. (1997). A comparison of acute mortality and population growth rate as endpoints of toxicological effect. Ecotoxicol. Environ. Saf..

[22] Mahdavi V., Saber M., Rafiee D.H., Mehrvar A. (2011). Comparative study of the population level effects of carbaryl and abamectin on larval ectoparasitoid *Habrobracon hebetor* Say (Hymenoptera: Braconidae). BioControl.

[23] Desneux N., Decourtye A., Delpuech J.M. (2007). The sublethal effects of pesticides on beneficial arthropods. Annu. Rev. Entomol..

[24] Wang D.S., He Y.R., Guo X.L., Luo Y.L. (2012). Acute toxicities and sublethal effects of some conventional insecticides on *Trichogramma chilonis* (Hymenoptera: Trichogrammatidae). J. Econ. Entomol..

[25] Moura R., Garcia P., Cabral S., Soares A.O. (2006). Does pirimicarb affect the voracity of the euriphagous predator, *Coccinella undecimpunctata* L. (Coleoptera: Coccinellidae)?. Biol. Control.

[26] Palumbo J.C., Horowitzb A.R., Prabhaker N. (2001). Insecticidal control and resistance management for *Bemisia tabaci*. Crop Prot..

[27] Tomizawa M., Casida J.E. (2003). Selective toxicity of neonicotinoids attributable to specificity of insect and mammalian nicotinic receptors. Annu. Rev. Entomol..

[28] Jeschke P., Nauen R. (2008). Neonicotinoids—From zero to hero in insecticide chemistry. Pest Manag. Sci..

[29] Zhao X.P., Wu C.X., Wang Y.H., Cang T., Chen L.P., Yu R.X., Wang Q. (2012). Assessment of toxicity risk of insecticides used in rice ecosystem on *Trichogramma japonicum*, an egg parasitoid of rice lepidopterans. J. Econ. Entomol..

[30] Sohrabi F., Shishehbor P., Saber M., Mosaddegh M.S. (2012). Lethal and sublethal effects of buprofezin and imidacloprid on the whitefly parasitoid *Encarsia inaron* (Hymenoptera: Aphelinidae). Crop Prot..

[31] Sohrabi F., Shishehbor P., Saber M., Mosaddegh M.S. (2013). Lethal and sublethal effects of imidacloprid and buprofezin on the sweetpotato whitefly parasitoid *Eretmocerus mundus* (Hymenoptera: Aphelinidae). Crop Prot..

[32] Biondi A., Zappala L., Stark J.D., Desneux N. (2013). Do biopesticides affect the demographic traits of a parasitoid wasp and its biocontrol services through sublethal effects?. PLoS ONE.

[33] Ohta I., Takeda M. (2015). Acute toxicities of 42 pesticides used for green peppers to an aphid parasitoid, *Aphidius gifuensis* (Hymenoptera: Braconidae), in adult and mummy stages. Appl. Entomol. Zool..

[34] Chitgar M.G., Ghadamyari M. (2012). Effects of Amitraz on the parasitoid *Encarsia formosa* (Gahan) (Hymenoptera: Aphelinidae) for Control of *Trialeurodes vaporariorum* Westwood (Homoptera: Aleyrodidae): IOBC Methods. J. Entomol. Res. Soc..

[35] Desneux N., Pham-Delègue M.H., Kaiser L. (2004). Effects of sub-lethal and lethal doses of lambda-cyhalothrin on oviposition experience and host-searching behaviour of a parasitic wasp, *Aphidius ervi*. Pest Manag. Sci..

[36] Cuthbertson A.G.S., Blackburn L.F., Northing P., Luo W., Cannon R.J.C., Walters K.F.A. (2009). Leaf dipping as an environmental screening measure to test chemical efficacy against *Bemisia tabaci* on poinsettia plants. Int. J. Environ. Sci. Technol..

[37] General Administration of Quality Supervision, Inspection and Quarantine of the People’s Republic of China and Standardization Administration of the People’s Republic of China (2014). Test Guidelines on Environmental Safety Assessment for Chemical Pesticides―Part 17: Trichogramma Acute Toxicity Test.

[38] De Morais M.R., Zanardi O.Z., Rugno G.R., Yamamoto P.T. (2016). Impact of five insecticides used to control citrus pests on the parasitoid *Ageniaspis citricola* Longvinovskaya (Hymenoptera: Encyrtidae). Ecotoxicology.

[39] Vanaclocha P., Vidal-Quist C., Oheix S., Monton H., Planes L., Catalan J., Tena A., Verdu M.J., Urbaneja A. (2013). Acute toxicity in laboratory tests of fresh and aged residues of pesticides used in citrus on the parasitoid *Aphytis melinus*. J. Pest Sci..

[40] Prabhaker N., Castle S.J., Naranjo S.E., Toscano N.C., Morse J.G. (2011). Compatibility of two systemic neonicotinoids, imidacloprid and thiamethoxam, with various natural enemies of agricultural pests. J. Econ. Entomol..

[41] Saber M. (2011). Acute and population level toxicity of imidacloprid and fenpyroximate on an important egg parasitoid, *Trichogramma cacoeciae* (Hymenoptera: Trichogrammatidae). Ecotoxicology.

[42] Suh C.P.C., Orr D.B., van Duyn J.W. (2000). Effect of insecticides on *Trichogramma exiguum* (Trichogrammatidae: Hymenoptera) preimaginal development and adult survival. J. Econ. Entomol..

[43] Preetha G., Stanley J., Suresh S., Kuttalam S., Samiyappan R. (2009). Toxicity of selected insecticides to *Trichogramma chilonis*: Assessing their safety in the rice ecosystem. Phytoparasitica.

[44] Wang H.Y., Yang Y., Su J.Y., Shen J.L., Gao C.F., Zhu Y.C. (2008). Assessment of the impact of insecticides on *Anagrus nilaparvatae* (Pang et Wang) (Hymenoptera: Mymanidae), an egg parasitoid of the rice planthopper, *Nilaparvata lugens* (Hemiptera: Delphacidae). Crop Prot..

[45] Hidrayani, Purnomo, Rauf A., Ridland P.M., Hoffmann A.A. (2005). Pesticide applications on Java potato fields are ineffective in controlling leafminers, and have antagonistic effects on natural enemies of leafminers. Int. J. Pest Manag..

[46] Mohammed A.A.H., Desneux N., Fan Y.J., Han P., Ali A., Song D.L., Gao X.W. (2017). Impact of imidacloprid and natural enemies on cereal aphids: Integration or ecosystem service disruption?. Entomol. Gen..

[47] Varenhorst A.J., O’Neal M.E. (2012). The response of natural enemies to selective insecticides applied to soybean. Environ. Entomol..

[48] Tran D.H., Takagi M., Takasu K. (2004). Effects of selective insecticides on host searching and oviposition behavior of *Neochrysocharis formosa* (Westwood) (Hymenoptera: Eulophidae), a larval parasitoid of the American serpentine leafminer. Appl. Entomol. Zool..

[49] Liu F., Bao S.W., Song Y., Lu H.Y., Xu J.X. (2010). Effects of imidacloprid on the orientation behavior and parasitizing capacity of *Anagrus nilaparvatae*, an egg parasitoid of *Nilaparvata lugens*. BioControl.

[50] Bethke J.A., Redak R.A. (1997). Effect of imidacloprid on the silverleaf whitefly, *Bemisia argentifolii* Bellows and Perring (Homoptera: Aleyrodidae), and whitefly parasitism. Ann. Appl. Biol..

[51] Desneux N., Rafalimanana H., Kaiser L. (2004). Dose-response relationship in lethal and behavioural effects of different insecticides on the parasitic wasp *Aphidius ervi*. Chemosphere.

[52] Bayram A., Salerno G., Onofri A., Conti E. (2010). Sublethal effects of two pyrethroids on biological parameters and behavioral responses to host cues in the egg parasitoid *Telenomus busseolae*. Biol. Control.

[53] Abbes K., Biondi A., Kurtulus A., Ricupero M., Russo A., Siscaro G., Chermiti B., Zappala L. (2015). Combined non-target effects of insecticide and high temperature on the parasitoid *Bracon nigricans*. PLoS ONE.

